# Temporal Patterns of Bacterial and Viral Communities during Algae Blooms of a Reservoir in Macau

**DOI:** 10.3390/toxins13120894

**Published:** 2021-12-13

**Authors:** Dini Hu, John P. Giesy, Min Guo, Wai Kin Ung, Yijun Kong, Kai Meng Mok, Simon Ming-Yuen Lee

**Affiliations:** 1 Department of Civil and Environmental Engineering, Faculty of Science and Technology, University of Macau, Macau SAR, China; hudini@bjfu.edu.cn (D.H.); kmmok@um.edu.mo (K.M.M.); 2 Key Laboratory of Non-Invasive Research Technology for Endangered Species, School of Ecology and Nature Conservation, Beijing Forestry University, Beijing 100083, China; 3 Department of Veterinary Biomedical Sciences and Toxicology Centre, College of Veterinary Medicine, University of Saskatchewan, Saskatoon, SK S7N5B3, Canada; jgiesy@aol.com; 4 School of Biological Sciences, University of Hong Kong, Hong Kong SAR, China; 5 State Key Laboratory of Pollution Control and Resource Reuse, School of the Environment, Nanjing University, Nanjing 210089, China; 6 State Key Laboratory of Quality Research in Chinese Medicine and Institute of Chinese Medical Sciences, University of Macau, Macau SAR, China; guomin5208@163.com; 7 Laboratory & Research Center, Macao Water Supply Co. Ltd., Conselheiro Borja, Macau SAR, China; kin.ung@macaowater.com (W.K.U.); edwards.kong@macaowater.com (Y.K.)

**Keywords:** phytoplankton, cyanobacteria, algal viruses, viral metagenomics, Asia, hazardous algal bloom

## Abstract

Compositions of microbial communities associated with blooms of algae in a storage reservoir in Macau, China were investigated between 2013 and 2016. Algae were enumerated by visible light microscopy. Profiles of organisms in water were examined by 16S rRNA sequences and viral metagenomics, based on next generation sequencing. Results of 16S rRNA sequencing indicated that majority of the identified organisms were bacteria closely related to Proteobacteria, Cyanobacteria, Verrucomicrobia, Bacteroidetes, and Actinobacteria. Metagenomics sequences demonstrated that the dominant virus was *Phycodnavirus*, accounting for 70% of the total population. Patterns of relative numbers of bacteria in the microbial community and their temporal changes were determined through alpha diversity indices, principal coordinates analysis (PCoA), relative abundance, and visualized by Venn diagrams. Ways in which the bacterial and viral communities are influenced by various water-related variables were elucidated based on redundancy analysis (RDA). Relationships of the relative numbers of bacteria with trophic status in a reservoir used for drinking water in Macau, provided insight into associations of *Phycodnavirus* and *Proteobacteria* with changes in blooms of algae.

## 1. Introduction

Due to inputs of excess nutrients during cultural eutrophication and temperature, blooms of freshwater algae and Cyanobacteria are prevalent in tropical and sub-tropical areas of Asia [1]. Primary producers in eutrophic lakes or reservoirs are often dominated by Cyanobacteria, formerly known as blue-green algae, which can produce various cyanotoxins including microcystins (*Cylindrospermopsis* and *Nodularia*), which adversely affect ecosystems and the economically and aesthetically important services they provide to humans [2]. Thus, it is important to understand compositions of microorganisms during algal blooms.

Freshwater ecosystems contain thousands of microorganisms, including bacteria, archaea, eukaryotes, and viruses, which together form complex microbial communities that influence ecosystem processes [3]. Studies of aquatic, microbial communities commonly focus on structure and function of populations to deduce fundamental factors that are associated with observed changes as functions of space and time [4]. In freshwater environments, physical, chemical, and biological reactions can differ profoundly and have significant effects on the overall quality of water [5]. Blooming of Cyanobacteria is related to eutrophication, which due to the agricultural and industrial activities of humans enhanced by urbanization, result in large amounts of key nutrients, primarily phosphorus (P) but also nitrogen (N), accumulating in aquatic ecosystems [6]. Results of previous studies have demonstrated that the nutrients P and N, as well as temperature were the primary factors controlling standing crop and rates of growth of populations of Cyanobacteria in eutrophic environments [7]. In addition to the bacterial communities, viruses which infect phytoplankton and Cyanobacteria are of particular importance and have been implicated as accessory factors causing or facilitating blooms of hazardous algae [8]. There are at least two kinds of virus that can infect algae. The first kind is the rod-shaped, single-stranded (ss) RNA Furoviruses, which are the only known RNA viruses that infect algae [9]. The other kind is the dsDNA viruses belonging to the family Phycodnaviridae that can infect protists and some brown algae [10]. To understand hazardous algal blooms and determine how to best control or manage them, it was deemed essential to explore relationships between viruses and their host bacteria and environmental factors determining communities in freshwater ecosystems.

The present study was conducted in the Macau Storage Reservoir (MSR) in Macau, SAR, China, which receives water from the West River, a channel of the Pearl River network, which is also eutrophied [11,12,13,14]. So far, most studies on diversity, distribution, absolute and relative abundances of phytoplankton taxa in MSR were done by microscopic examination and identifications based on morphological characteristics [15,16]. Therefore, many less abundant, hazardous algae were not accurately detected by the methods of microscopic identification and enumeration, the phytoplankton composition in MSR was not fully understood [17]. Currently, most studies on compositions of communities of microbes in freshwater environments using high-throughput sequencing techniques have shown advantages in describing microbial communities [18,19,20]. Compositions of communities of microbes in aquatic environments have been studied based on 16S rRNA sequences [21,22,23]. In addition, metagenomics is a method that can be used to reveal unknown viruses and to describe diversity for more comprehensive understanding of aquatic environments [24]. Thus, sequencing depth can be combined with viral metagenomics and 16S rRNA sequences to investigate relationships between viruses and their various hosts.

In this study, MSR was selected for exploring the microbial communities and their interactions in a freshwater ecosystem. The main goals were to: (1) determine composition of the bacterial communities in the reservoir based on 16S rRNA sequences; (2) explore diversity of the virus community and its potential relationships with the hosts by metagenomics sequences under a range of physicochemical conditions; (3) investigate the pattern of, and possible factors that affect, temporal changes in the microbial community; and (4) analyze the relationships between structures of microbial communities and water-related variables during blooms of harmful Cyanobacteria. Since the water treatment industry has traditionally been focusing on evaluating the adverse effects of microbial communities on public health, results of the present study could provide additional information on biodiversity and the structure of communities of microorganisms in freshwater environments during blooms of Cyanobacteria, which could assist in assessing risks to humans.

## 2. Results

### 2.1. Algal Biomass and Temporal Changes

**Figure 1:**
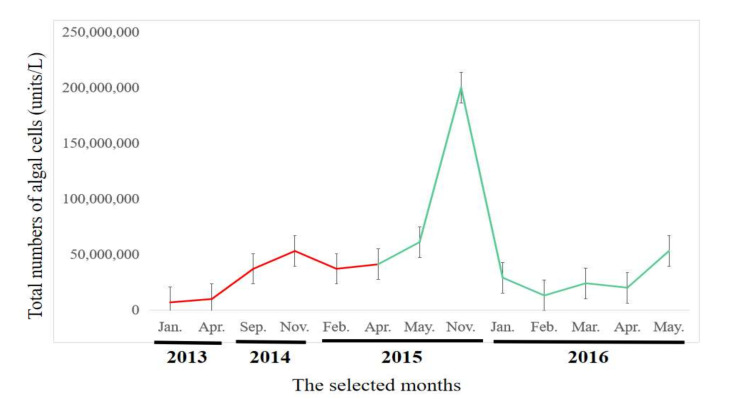
Total numbers of algal cells (units/L) in Macau Storage Reservoir (MSR) from 2013 to 2016. Error bars represent standard deviations (SD).

In recent years, MSR have experienced severe algal blooms (Supplementary Figure S1). Numbers of algal cells remained stable at about 6.8 × 10^6^ to 6.1 × 10^7^ units/L throughout the selected months and had a significant increase on November 2015 (Figure 1).

### 2.2. 16S rRNA Sequencing Results and Diversity Analysis

A total of 573,863 raw 16S rRNA V4 sequencing reads from the 13 samples were subjected to Illumina sequencing. A total of 229,838 reads remained after passing through quality control and having chimeras removed and were clustered into 30,213 operational taxonomic units (OTUs) detection.

Sequences were grouped into OTUs to compare diversities in species richness. The whole phylogenetic diversity tree (PD whole tree) and Chao 1 were used to investigate species richness. Alpha diversity, which refers to biodiversity within communities, was represented by Shannon and Simpson diversity indices. Mean values of the PD whole tree, Chao 1, observed species, and Shannon and Simpson indices of all samples were 105.46, 3765.99, 1435.00, 7.45, and 0.96, respectively (Supplementary Table S1), which represented relatively great species and community diversity. Chao 1 and OTUs reflect the richness of the community, estimated for all species within a community, and the value of Chao 1 and number of OTUs indicated that species of this ecosystem were abundant in the sample.

### 2.3. Taxonomic Compositions of Bacterial Communities

**Figure 2:**
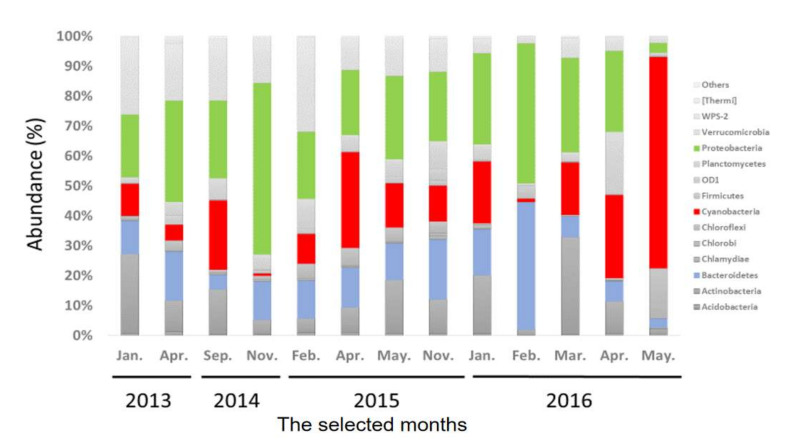
Taxonomic composition based on the 16S rRNA sequences, of the bacterial communities at the phylum level.

**Figure 3:**
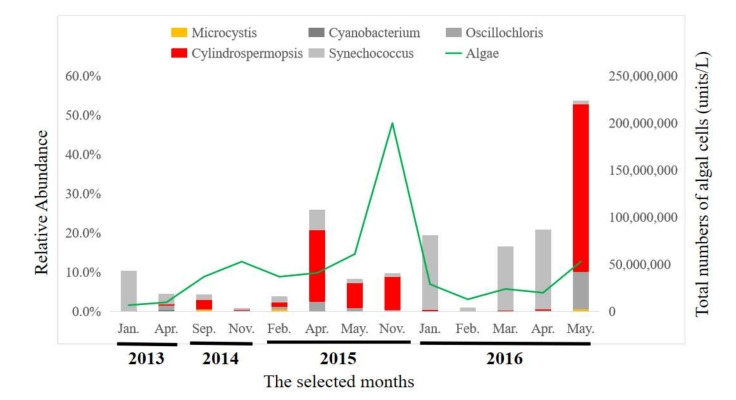
Taxonomic composition, based on the 16S rRNA sequences, of the Cyanobacterial communities at the genus level.

To understand the temporal dynamics of bacterial communities in MSR, monthly random samples of water were collected during the study period. Diverse communities of bacteria were observed at the level of phylum (Figure 2). Even though there were slight differences among months, proteobacteria was the dominant phylum for all samples and contributed approximately 32% of the total OTU, followed by Cyanobacteria (18.9%), *Bacteroidetes* (14.1%), and *Verrucomicrobia* (11.5%). The 10 most prevalent phyla were Proteobacteria, Cyanobacteria, Bacteroidetes, Verrucomicrobia, Actinobacteria, Planctomycetes, Chloroflexi, OD1, Acidobacteria, Firmicutes, Chlorobi, Chlamydae, WPS-2, and others. Cyanobacteria represented the greatest proportion of phytoplanktonic communities in the detected bacterial community, which were composed primarily of species from five genera of Cyanobacteria, *Cylindrospermopsis*, *Synechococcus*, *Oscillochloris*, *Microcystis*, and *Cyanobacterium* (Figure 3). *Microcystis* and *Cylindrospermopsis* produce toxic extracellular products (Supplementary Figures S2 and S3) [25].

Seasons in Macau were defined as follows: spring in March and April, summer from May to September, autumn from October to December and winter in January and February. The phylum Cyanobacteria was more abundant during spring and summer. The toxic Cyanobacterium, *Cylindrospermopsis* was more abundant in April and May, but was less abundant during winter. *Microcystics* occurred at a relatively lesser abundance during all seasons.

### 2.4. Composition of Viral Communities Based on Metagenomic Sequences

**Figure 4:**
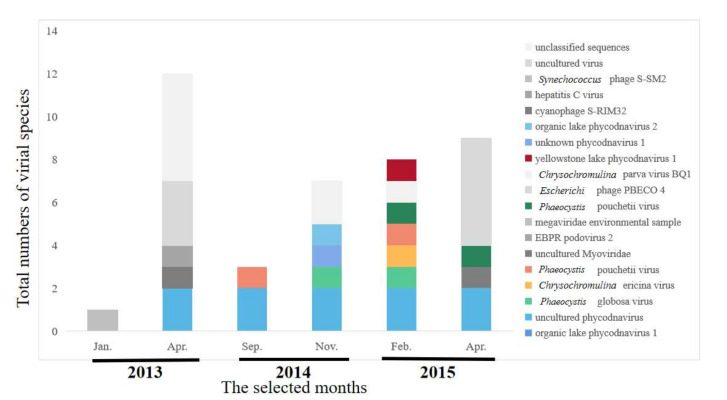
Abundances of viral species based on metagenomic sequences of viruses in MSR.

Six months of samples were selected to identify the viral communities, during which amounts of algae were relatively stable (Figure 1, highlighted in red). A total of 24,973,442 raw metagenomics reads were obtained, from which 1621 contigs were generated (>90 bp each) during the 6 months of MSR monitoring. The dominant family of viruses was Phycodnaviridae, which accounted for 70% of the total. Phycodnaviridae is considered to survive with algae as a host. Other viruses belonged to typical bacteriophage families, including: Myoviridae, Podoviridae, Flaviviridae, and Miniviridae. Metagenomics sequences were used to identify constituents of microbial communities at the species level of classification (Figure 4). The primary viruses included Organic lake *Phycodnavirus* 2, an unidentified *Phycodnavirus* 1, Yellowstone lake *Phycodnavirus* 1, *Phaeocystis pouchetii* virus 1, *Phaeocystis pouchetii* virus, *Phaeocystis globosa* virus, and uncultured *Phycodnavirus,* which belongs to the family Phycodnaviridae. The richness of viral species were significantly greater in spring, with values of 13.04 and 16.04 compared to those during other seasons (Figure 4). The compositions of viral species in the communities exhibited temporal variations. The greatest richness of species composition was found in spring. Species belonging to the Phycodnaviridae were abundant during autumn and winter.

### 2.5. Relationships between Phycodnavirus and Communities of Algae and Bacteria

**Figure 5:**
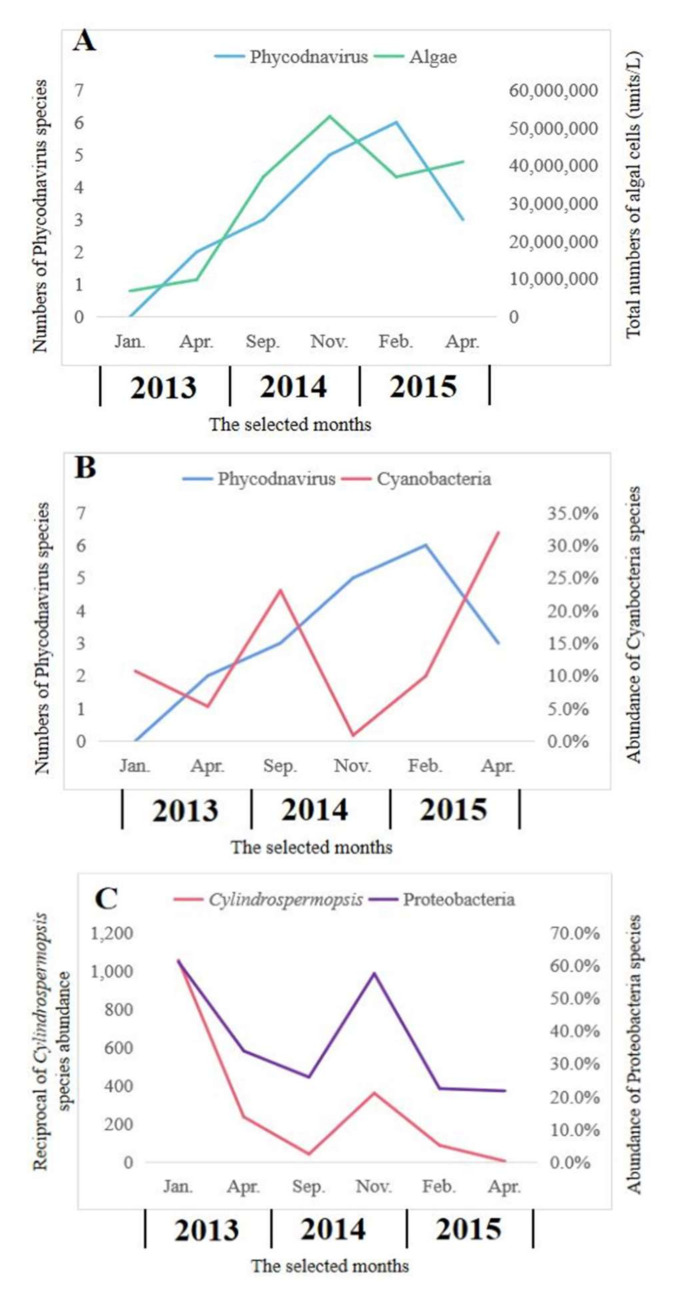
Relationships between Phcodnavirus and algae (**A**), Phycodnavirus and Cyanobacteria (**B**) and Proteobacteria and toxic Cyanobacteria of *Cylindrospermopsis* (**C**).

Viruses that can affect phytoplankton have been studied for many years. Most of them belong to the family Phycodnaviridae. An important but challenging aspect of algal viruses is the understanding of the relationship with their hosts. *Phycodnavirus* and algae exhibited similar seasonal trends in abundances, as the peak of algae occurred before the peak of viruses (Figure 5A). There was a statistically significant (R^2^ = 0.640, *p* = 0.031) relationship between the numbers of algae and *Phycodnavirus*. However, there was no statistically significant (R^2^ = 0.001, *p* = 0.962) relationship between the numbers of Cyanobacteria and numbers of *Phycodavirus* (Figure 5B). In this study, the dominant Proteobacteria exhibited a significant relationship with *Cylindrospermopsis*, which belonged to the toxic *Cyanobacteria* (R^2^ = 0.812, *p* = 0.009) (Figure 5C).

### 2.6. Comparison of Microbial Community Composition with Water Parameters

**Figure 6:**
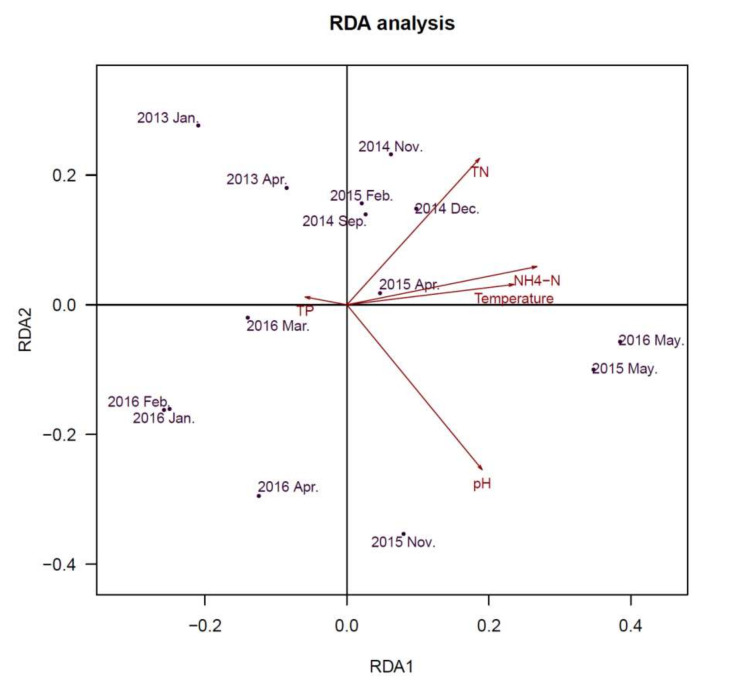
Redundancy analysis (RDA) ordination diagram of Cyanobacterial community at the genus level, with environmental variables as arrows. Circles represent various samples. Samples are ordinated in relation to the environmental variables with which they have the strongest association.

**Figure 7:**
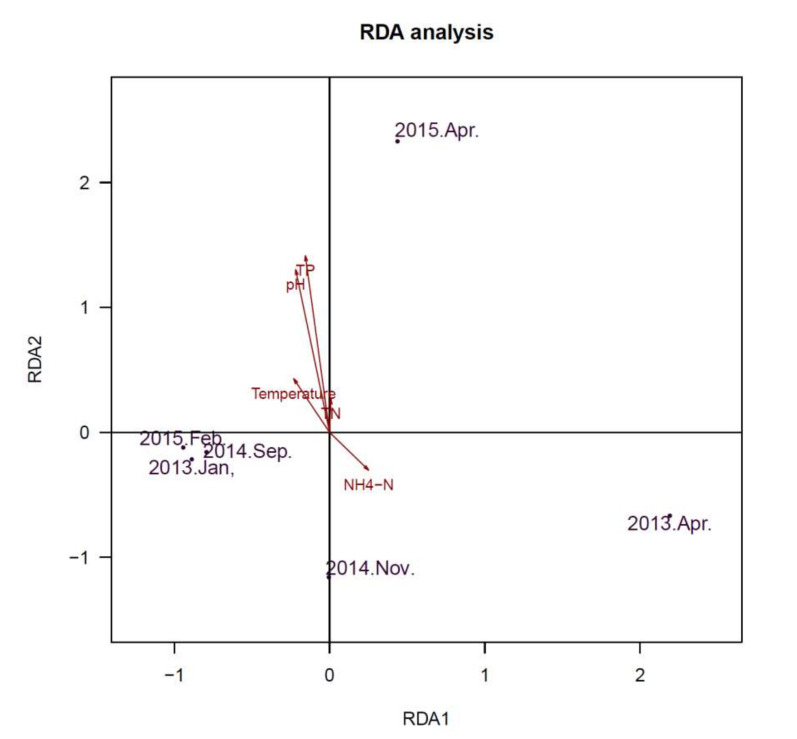
Redundancy analysis (RDA) ordination diagram of the viral community at the species level, with environmental variables as arrows. Circles represent various samples. Samples are ordinated in relation to the environmental variables with which they have the strongest association.

Redundancy Analysis (RDA) was used to determine whether Cyanobacterial community and viral communities were related to environmental variables. The axes (Figure 6) represent the strongest relationships between gradients in microbial community composition and measured environmental variables, which indicate that variations of environmental variables determined variations in the constituents of the community. Water parameters of total nitrogen (TN), NH_4_-N, pH and temperature were significantly associated with the Cyanobacterial community (Figure 6). For the viral community (Figure 7), pH and concentration of TP exhibited significant associations. pH exhibited a positive relationship with axis 2, while total phosphorus (TP) were also positively correlated with axis 2. These results indicated that pH and concentration of TP (TN/TP) were associated with microbial community in this freshwater ecosystem.

## 3. Discussion

In-depth bioinformatics analyses based on 16S rRNA sequences provide greater insight into species compositions. In this study, an average of 44,143 reads per sample allowed identification of a total of 30,213 OTUs among 30 phyla in 13 samples of water collected from a freshwater reservoir. Results indicate that communities were diverse and dominated by bacteria rather than archaea or other microbes. Proteobacteria, Verrucomicrobia, Bacteroidetes, Actinobacteria, and Cyanobacteria were the dominant phyla. This finding is consistent with those of previous studies, in which the major bacterial community could be classified into Alpha-proteobacteria, Beta-proteobacteria, Bacteroides, Actinobacterial, and Verrucomicrobia groups in most freshwater environments [26], while Proteobacteria dominated freshwater bacterial communities [27]. Results reported here were also consistent with those reported previously [28], which suggested that some bacterial groups, including Proteobacteria, Actinobacteria, and Verrumicrobia, were closely associated with toxic algal blooms [29]. Results of the current study also showed that Cyanobacteria was abundant during spring and summer; but there were also several genera of Actinobacteria. Similar results also have been observed in most studies. Although Actinobacteria was ubiquitous and dominant in freshwater systems, it was less abundant in nutrient-rich environments [30].

Although OTUs observed in the study had broad taxonomic diversity, only a small number of species exhibited significant changes with time. Cyanobacteria partially dominated the bacterial community in nutrient-rich freshwater environments during summer and spring, but are normally less abundant during winter. This finding was not unique; similar examples of species-specific bacterial colonization of aquatic systems have been observed [31]. In addition, Actinobacteria and Proteobacteria groups dominated more eutrophic aquatic ecosystems during winter when Cyanobacteria were lacking [32], similar result was also observed in this study. All of these findings suggested that core bacterial communities can occupy environments [33]. However, this does not mean that other groups are not present at significant abundances during some seasons. Although specific environmental conditions were created by Cyanobacterial communities, they did not lessen the richness of non-Cyanobacteria populations, and a large number of bacteria not associated with algae were present in characteristic of eutrophic freshwater environments [29].

High-throughput sequencing revealed a bacterial community of great diversity, as well as algal viruses. In the MSR, *Phycodanvirus*, which can survive in the algae as their host, were observed in various algae. Results of this and other studies demonstrate that high-throughput sequencing is a powerful tool for detecting the microbial community to help understanding the interactions between algae and microbes in freshwater ecosystems [34]. The number of *Phycodnavirus* was related with the total number of algal cells (R^2^ = 0.640, *p* = 0.031). *Phycodnavirus* has been widely recognized to infect algae [35], but in this study, no significant (R^2^ = 0.001, *p* = 0.962) association was observed between *Phycodnavirus* and algal cells. However, the dominant bacteria, Proteobacteria, exhibited a significant relationship with Cyanobacteria including even hazardous Cyanobacteria such as *Cylindrospermopsis* (R^2^ = 0.812, *p* = 0.009). Up to now, there is no study on the association between the growth of the Proteobacteria and toxic Cyanobacteria. The exact mechanism of their interaction remains unclear and needs to be further elucidated.

Furthermore, a cyclic pattern of relative abundances of microorganisms was observed in this study with Cyanobacteria mainly accumulated during spring and summer, which is consistent with results of other studies where significant microbial populations accumulated in a single season [36]. While microorganisms experience strong selection pressure under extreme conditions, they can still be clustered together, which illustrated that the survival of the species are strongly dependent on environmental conditions [37]. Alternatively, chemical and physical conditions can also drive changes in absolute and relative members of the microbial community over time [38]. Many of the microorganisms observed during this study had coexisted in this environment for a long time, and they also participate in a variety of physical and chemical reactions; therefore, environmental conditions can lead to changes in structure of the microbial community [39]. Based on results of the RDA analysis, pH and concentration of TP (TN/TP) were associated with the microbial community. This observation is in agreement with the results of other studies that showed influences of environmental factors [40]. Previous analyses revealed a significant correlation between structures of microbial communities and environmental factors, with pH being the most important. This is consistent with results found based on 16S rRNA gene sequences in previous studies, that pH was one of the most influential factors in structuring microbial communities [30,41]. In addition to pH, temperature also plays a role in shaping structures of communities of microorganisms [42]. Several previous studies have demonstrated that changes in temperature affect metabolism, production, respiration, and the growth efficiency of bacterial communities [43]. Another significant factor affecting bacterial communities during different seasons is organic matter, which has a strong impact on metabolism of microbes [44].

Based on results of this study, it can be concluded that specific compositions of bacterial and viral communities that reflected the characteristics of a eutrophic freshwater environment were observed. Meanwhile, the microbial community was structured by a series of environmental factors. The critical factor was that *Phycodnavirus* and Proteobacteria affect both algae and hazardous Cyanobacteria. This information is important to the water utilities for developing monitoring strategies to detect the algal blooms in raw water sources as well as the treatment strategies that can minimize the adverse effects of algae. However, to understand why the numbers of algae increased significantly in November 2015, further studies are needed.

## 4. Materials and Methods

### 4.1. Sampling Sites and Collection

**Figure 8:**
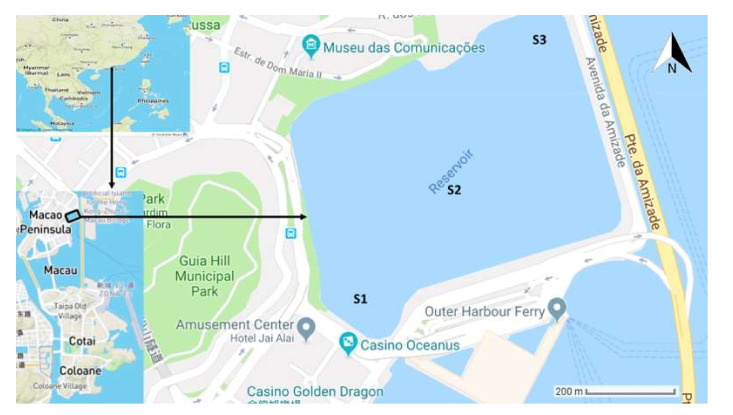
Sampling locations in Macau Storage Reservoir (MSR).

The MSR (22°12′12″ N, 113°33′12″ E) is a freshwater storage unit, with a capacity of 1.9 million m^3^, which is located in Macau, SAR, China, on the western side of the Pearl River Delta across Hong Kong (Figure 8). This region is characterized by a humid, subtropical climate, with the seasonal climate being influenced by summer and winter monsoons. This reservoir has been experiencing eutrophication with blooms of Cyanobacteria which, in recent years, seem to be worsening. The dominant actinobacterial species were *Microcystis aeruginosa* and *Cylindrospermopsis raciborskii* [45]. The phytoplankton abundance was between 10 and 150 million cells/L during one year [46].

The water samples were collected during 2013–2016. Three sampling points were selected at three stations S1–S3 (Figure 8) in the reservoir. Stations S1, S2, and S3 are located in the inlet, center, and outlet of the reservoir, respectively. Samples were collected from 0.5 m below the surface at each station. Waters collected from S1, S2, and S3 were homogenized to represent one composite sample for each month. Samples were kept at −80 °C prior to analysis and extraction of DNA. Samples from January 2013, April 2013, September 2014, November 2014, February 2015, April 2015, May 2015, November 2015, January 2016, February 2016, March 2016, April 2016, and May 2016 were used for 16S rRNA sequencing. Samples of January 2013, April 2013, September 2014, November 2014, February 2015 and April 2015 were used for metagenomics sequencing. Macau Water Supply Co. Ltd. is responsible for monitoring and management of water-quality in the reservoir, which is the source of drinking water for Macau.

### 4.2. Water Parameters

Samples were analyzed for five abiotic parameters. The temperature of water was measured in situ by a mercury thermometer. The pH was measured in the laboratory with a pH meter (DKKTOA, HM-30R, Tokyo, Japan). Ammonia-nitrogen (NH_4_–N), total nitrogen (TN) and total phosphorus (TP) were measured by Nessler’s reagent and spectrophotometry [47], flow injection analysis-spectrophotometry [48] and persulfate oxidation [49], respectively. In addition to abiotic parameters, the total number of algae was enumerated through an inverted light microscope [50].

### 4.3. Extraction and Sequencing of DNA

On each date of collection, samples of water were collected from three sites, and mixed into a single composite sample to provide an integrated, representative assessment of species diversity. One liter of samples of water were filtered through polycarbonate membrane filters (pore size 0.22 μm, diameter 73 mm, Millipore, Burlington, MA, USA), then the filtered water was passed through another smaller membrane (pore size 0.10 μm, membrane diameter 91 mm, JET BIOFIL, Guangzhou, China). The membranes were rolled and inserted into a Power-water Bead Tube (Mo Bio Laboratories Inc., Carlsbad, CA, USA). DNA was extracted from membranes by a PowerWater^®^ DNA isolation kit (Mo Bio Laboratories Inc., Carlsbad, CA, USA) according to the manufacturer’s protocol.

Data on DNA sequences were generated based on the Illumina (San Diego, CA, USA) sequencing platform using bacteria primers 515F/806R, which target the V4 region of bacterial 16S rRNA genes. Sequences need to be processed preliminarily for quality control, including checks of guanine-cytosine (GC) percentage distribution and overrepresented sequences. Observed species richness values were clustered at 97% similarity to define a bacterial species at the V4 region of 16S rRNA genes. Metagenomic sequencing was performed on Hiseq2500 platform (Illumina, San Diego, CA, USA).

### 4.4. Data Analysis

FastQC (version 0.11.9, Illumina, San Diego, CA, USA) was used to evaluate quality of sequencing, and raw reads were obtained from the sequencer. After assessment of qualities of raw sequence reads, low quality reads were removed through the Trimmonatic algorithm (http://www.usadellab.org/cms/index.php?page=trimmomatic, accessed on 17 November 2021) to obtain the final set of data. Mothur 1.35 (https://www.mothur.org/wiki/Download_mothur, accessed on 17 November 2011) and Flash 1.2.1 (http://ccb.jhu.edu/software/FLASH/, accessed on 17 November 2021) were used to trim sequences and remove chimera sequences to obtain raw contigs. Sequences were assigned using QIIME (http://qiime.org/install/index.html, accessed on 17 November 2021), based on information on barcodes and primers. VirusTAP (https://gph.niid.go.jp/virustap/system_in, accessed on 17 November 2021) was used to do de novo assembly of the genomes of viruses.

Rarified operational taxonomic unit (OTU) tables were conducted to generate alpha diversity indices based on the whole phylogenetic diversity tree, Chao 1, observed species, Shannon indices, and Simpson indices. To further understand community compositions, R software (https://www.datavis.ca/R/, accessed on 17 November 2021) was used to generate the principle coordinates analysis (PCoA) and the redundancy analysis (RDA) figure. RDA was used to determine significances of differences among groups and to identify which environmental parameters related to changes in compositions of bacterial and viral communities. Regression analysis was conducted in SPSS Statistics 23 (IBM, Armonk, NY, USA).

## Data Availability

Not applicable.

## Supplementary Materials

The following are available online at https://www.mdpi.com/article/10.3390/toxins13120894/s1, Figure S1: Total numbers of algal cells (units/L) in Macau Storage Reservoir (MSR) from 2013 to 2016, Figure S2: The microscopy photo of Microcystis, Figure S3: The microscopy photo of Cylindrospermopsis, Table S1: The values of PD whole tree, Chao 1, observed species, shannon and simpson indices of selected samples.
