# Supplementary material for: Temporal Patterns of Bacterial and Viral Communities during Algae Blooms of a Reservoir in Macau
Source: Toxins (Basel). 2021 Dec 13;13(12):894. doi: 10.3390/toxins13120894 (PMC8704429; doi:10.3390/toxins13120894)
Supplement: Supplementary file 1 [file toxins-13-00894-s001.zip › toxins-1436018-supplementary.pdf]

toxins

MDPI

Supplementary Information

Temporal Patterns of Bacterial and Viral Communities during Algae Blooms of a Reservoir in Macau

Dini Hu [1,2], John P. Giesy [3,4,5], Min Guo [6], Wai Kin Ung [7], Yijun Kong [7], Kai Meng Mok [1] and Simon Ming-Yuen Lee [6,*]

1. Department of Civil and Environmental Engineering, Faculty of Science and Technology, University of Macau, Macau SAR, China; hudini@bjfu.edu.cn (D.H.); kmmok@um.edu.mo (K.M.M.)
2. Key Laboratory of Non-Invasive Research Technology for Endangered Species, School of Ecology and Nature Conservation, Beijing Forestry University, Beijing 100083, China
3. Department of Veterinary Biomedical Sciences and Toxicology Centre, College of Veterinary Medicine, University of Saskatchewan, Saskatoon, SK S7N5B3, Canada; jgiesy@aol.com
4. School of Biological Sciences, University of Hong Kong, Hong Kong SAR, China
5. State Key Laboratory of Pollution Control and Resource Reuse, School of the Environment, Nanjing University, Nanjing 210089, China
6. State Key Laboratory of Quality Research in Chinese Medicine and Institute of Chinese Medical Sciences, University of Macau, Macau SAR, China; guomin5208@163.com
7. Laboratory & Research Center, Macao Water Supply Co. Ltd., Conselheiro Borja, Macau,SAR, China; kin.ung@macaowater.com (W.K.U.); edwards.kong@macaowater.com (Y.K.)

* Correspondence: simonlee@um.edu.mo

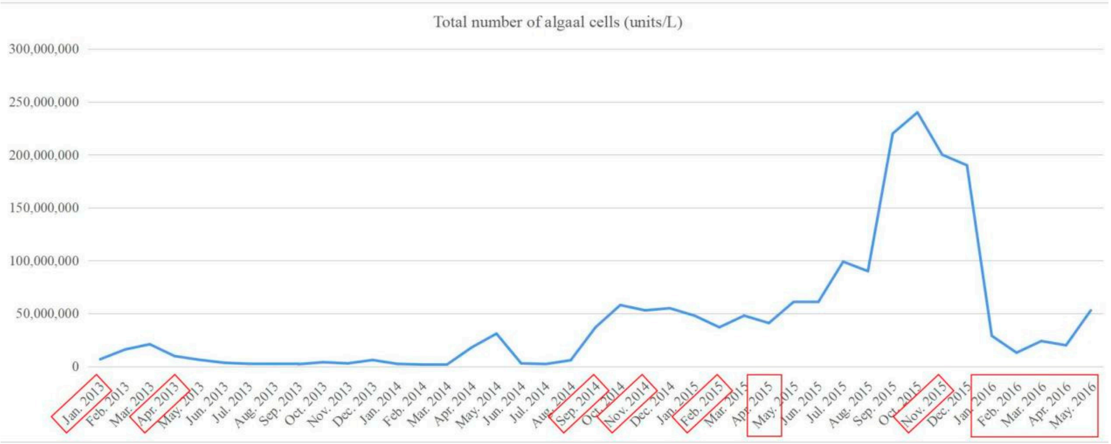

Figure S1. Total numbers of algal cells (units/L) in Macau Storage Reservoir (MSR) from 2013 to 2016.

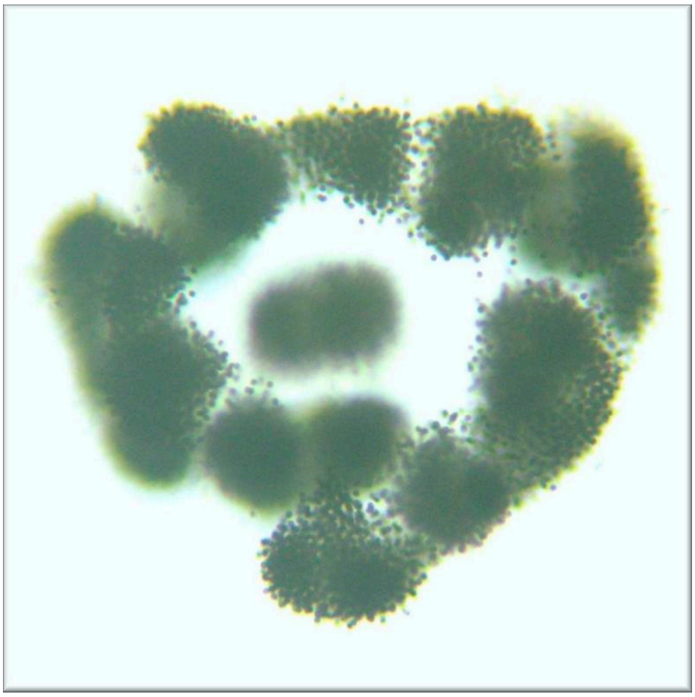

Figure S2. The microscopy photo of *Microcystis*.

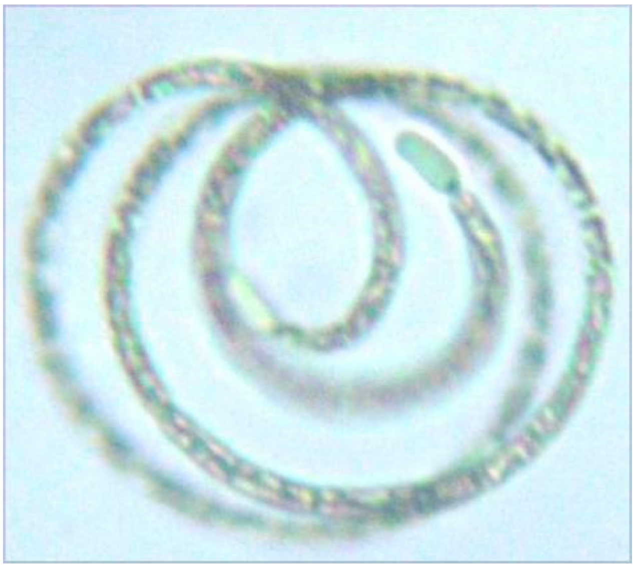

Figure S3. The microscopy photo of *Cylindrospermopsis*.

Table S1. The values of PD whole tree, Chao 1, observed species, shannon and simpson indices of selected samples.

	**PD Whole Tree**	**Chao1**	**Observed Species**	**Shannon**	**Simpson**
Jan. 2013	106.00	3699.91	1580.00	7.68	0.97
Apr. 2013	127.00	5228.34	1718.00	7.86	0.97
Sep. 2014	110.00	3585.52	1441.00	7.36	0.96
Nov. 2014	91.00	2704.88	1101.00	6.74	0.95
Feb. 2015	115.00	4218.09	1628.00	7.75	0.97
Apr. 2015	109.00	3880.41	1424.00	7.45	0.97
May. 2015	111.00	4119.54	1419.00	7.53	0.98
Nov. 2015	119.00	4301.21	1490.00	8.02	0.98
Jan. 2016	113.00	3979.99	1486.00	7.81	0.98
Feb. 2016	82.00	3205.30	1373.00	7.56	0.97
Mar. 2016	116.00	4423.81	1728.00	8.04	0.97
Apr. 2016	101.00	3548.72	1312.00	7.24	0.97
May. 2016	71.00	2062.09	955.00	5.82	0.89
Mean value	105.46	3765.99	1435.00	7.45	0.96
